# Surfactant protein B gene variations enhance susceptibility to squamous cell carcinoma of the lung in German patients

**DOI:** 10.1038/sj.bjc.6600353

**Published:** 2002-07-02

**Authors:** C Seifart, U Seifart, A Plagens, M Wolf, P von Wichert

**Affiliations:** Department of Internal Medicine, Division of Respiratory and Critical Care Medicine, Philipps-University of Marburg, Baldingerstraße, 35043 Marburg, Germany; Department of Internal Medicine, Division of Oncology and Hematology Philipps-University of Marburg, Baldingerstraße, 35043 Marburg, Germany

**Keywords:** lung cancer, surfactant, squamous cell carcinoma, SP-B, polymorphism

## Abstract

Genetic factors are thought to influence the risk for lung cancer. Since pulmonary surfactant mediates the response to inhaled carcinogenic substances, candidate genes may be among those coding for pulmonary surfactant proteins. In the present matched case–control study a polymorphism within intron 4 of the gene coding for surfactant specific protein B was analysed in 357 individuals. They were divided into 117 patients with lung cancer (40 patients with small cell lung cancer, 77 patients with non small cell lung cancer), matched controls and 123 healthy individuals. Surfactant protein B gene variants were analysed using specific PCR and cloned surfactant protein B sequences as controls. The frequency of the intron 4 variation was similar in both control groups (13.0% and 9.4%), whereas it was increased in the small cell lung cancer group (17.5%) and the non small cell lung cancer group (16.9%). The gene variation was found significantly more frequently in patients with squamous cell carcinoma (25.0%, *P*=0.016, odds ratio=3.2, 95%CI=1.24–8.28) than in the controls. These results indicate an association of the surfactant protein B intron 4 variants and/or its flanking loci with mechanisms that may enhance lung cancer susceptibility, especially to squamous cell carcinoma of the lung.

*British Journal of Cancer* (2002) **37**, 212–217. doi:10.1038/sj.bjc.6600353 www.bjcancer.com

© 2002 Cancer Research UK

## 

Individual cancer risk is due to gene-environmental interaction. Exposure to exogenous carcinogens, such as tobacco smoke is clearly related to the development of lung cancer. However, genetic factors may strongly mediate the risk among those who are exposed to carcinogens, as only 11% of the tobacco smokers ultimately develop lung cancer (Amos *et al*, 1999). In patients with impaired lung function, incidence and mortality of lung cancer is increased, independent of smoking habits, age and family history (Skillrud *et al*, 1986). Thus, in response to environmental mutagens the individual variability may be mediated by local factors of the airways.

The pulmonary surfactant system is essential for normal lung function and play an important role in mediating local airway condition. Pulmonary surfactant is a complex mixture of lipids, primarily phospholipids, and surfactant specific proteins. Structure and homeostasis of the system depends on the surfactant specific proteins SP-A, B and C. Next to surface tension lowering properties at the air/liquid interface, a number of additional functions have been attributed to the surfactant system. A bronchiolar surfactant layer has been demonstrated (Morgenroth, 1985) that appears to be important for several physiological functions of the airways. It improves bronchiolar mucociliary clearance, encoates inhaled particles and substances and is involved in their clearance from the upper respiratory tract (Gehr *et al*, 1993; De Sanctis *et al*, 1994). Because of the importance of surfactant in normal lung function, in mediating local airway conditions and in the clearance of the upper respiratory tract, surfactant protein genes may be good candidates in the study of etiologic factors for lung cancer.

Surfactant protein B is important for the formation of the active surfactant surface film (Schürch *et al*, 1998) and is essential for normal lung function (Robertson *et al*, 1991). Surfactant protein B knock out mice show disruption of surfactant film and function and die from respiratory failure (Clark *et al*, 1995, 1997). Furthermore, these mice are more susceptible to oxidative lung injury (Tokieda *et al*, 1999).

The gene for SP-B has been localized on the short arm of chromosome 2 (Emrie *et al*, 1988; Vamvakopoulos *et al*, 1995), consisting of 11 exons with the first 10 encoding the precursor protein. Different polymorphisms within the SP-B gene have been described (Pilot-Matias *et al*, 1989; Nogee *et al*, 1994; Floros *et al*, 1995; Lin *et al*, 1998, 1999a) and SP-B gene variations have been associated with several diseases, such as respiratory distress syndrome (RDS) (Floros *et al*, 1995), COPD (Seifart *et al*, 2002) and congenital alveolar proteinosis (Nogee *et al*, 1994; Lin *et al*, 1998). Recently, an aberrantly spliced SP-B mRNA had been associated with lung disease (Lin *et al*, 1999a).

Genetic factors are important in the development of lung cancer and pulmonary surfactant may be involved in mechanisms that enhance cancer susceptibility in the lung. Therefore, we hypothesised that surfactant protein gene variants may be related to an increased risk for developing lung cancer. Because SP-B is essential for spreading of surfactant, we analysed a large gene variation within intron 4 of the surfactant protein B gene in a case–control study in patients with small cell lung cancer (SCLC) and non small lung cancer (NSCLC), their matched control and healthy individuals.

The data revealed an enhanced frequency of intron 4 variants in the disease groups, and a significantly higher frequency in the squamous cell carcinoma group, suggesting an association between the SP-B locus and/or its flanking loci and lung cancer susceptibility.

## METHODS

### Patients

We studied the frequency of a variant locus within intron 4 of the gene coding for the surfactant specific protein B in 357 Caucasian individuals. These were divided into two patient groups (non small cell lung cancer and small cell lung cancer), their matched controls and 123 healthy individuals. All patients suffering from lung cancer, who were treated between January 1999 and December 1999 in the Department of Oncology were included. A standard questionnaire was used to obtain information about smoking, age, and case history. To set up the matched pairs, from a total of 600 patients undergoing lung function testing in the Department of Internal Medicine those without pulmonary disease, matching age, gender and smoking habits were included. Of the 117 patients with lung cancer, 77 suffered from non small cell lung cancer (NSCLC) and 40 patients from small cell lung cancer (SCLC). Ten pack years (p/y) were used as cut-off for smoking classification, because a significant excess risk of 70% has been associated with every 10 pack years in a European multicentre study (Agudo *et al*, 2000). Smoking habits were classified as follows: (a) never smokers and ever smokers, who had smoked at least 100 cigarettes in his or her life time, but smoked less than 10 pack years, were classified as light smokers (b) former smokers stopped smoking at least 1 year before inclusion into the study, but smoked more than 10 pack years, were classified as former smokers and (c) current smokers smoked more than 10 pack years were classified as current smokers. Calculation of exact pack years were done for the former and current smokers of the lung cancer groups. The characteristics of the groups are summarised in Table 1A

**Table 1A:**
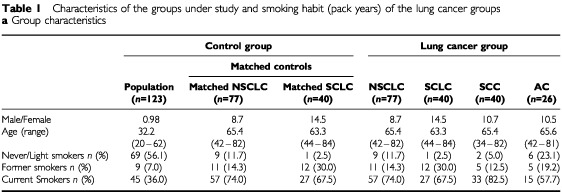
Characteristics of the groups under study and smoking habit (pack years) of the lung cancer groups**a** Group characteristics

and B

**Table 1B:**
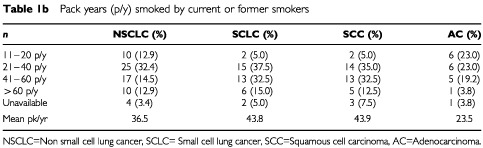
Pack years (p/y) smoked by current or former smokers

, and described below. Blood specimens were drawn for genotype analysis with informed consent of the patients.

### Population group

This group consisted of 123 healthy individuals. These individuals got physical examination by the staff medical service at the University of Marburg upon entering a new position or changing positions between October 1998 and July 1999. Age of the 123 individuals, 62 males and 61 females, ranged from 20 to 62 years. Forty-five (36.6%) individuals were classified as current smokers, nine (7.3%) as former smokers and 69 (56.1%) were never smokers or ever smokers smoked less than 10 pack years. Because the population control should represent a group comparable to the normal population the cases came from, the individuals are not age, smoking-habits, and gender matched. Therefore, the control population has a younger age than the patients and their matched controls.

### NSCLC-group

The NSCLC-group consisted of 77 patients with non small cell lung cancer, including 40 patients with squamous cell carcinoma (SCC), 26 with adenocarcinoma, five with large cell carcinoma and six with other tumour histology. As is typical of lung cancer, the group consisted of 69 males and eight females (gender-ratio: 8.6), with mean age 65.4 (42–82 years). Out of 77 patients, nine (11.7%) were never or light smokers, 11 (14.3%) were former smokers, while 57 (74.0%) were current smokers (Table 1A ,B). Six patients with adenocarcinoma, two with squamous cell carcinoma and one with undifferentiated carcinoma were classified as light smokers. Ten patients had not smoked more than 20 p/y (12.9%), 25 patients smoked 21–40 p/y (32.4%), 17 patients smoked 41–60 p/y by (14.5%) and 10 patients smoked more than 60 p/y (12.9%). Of four patients exact calculation of p/y was not possible. Mean of p/y was 36.5 for all NSCLC patients, 43.9 for the SCC-, and 23.5 for AC-subgroup.

### SCLC-group

Forty patients with small cell lung cancer were included. As common for SCLC, this group consisted of 35 males and five females (gender-ratio: 7.0). Patients age ranged from 44–84, the mean age was 63.3. One patient was not classified as a current or former smoker. Two patients (5.0%) had not smoked more than 20 p/y, 15 (37.5%) between 21–40 p/y, 13 (32.5%) between 41–60 p/y and six patients (15.0%) smoked more than 60 p/y (Table 1A,B). Mean number of p/y was 43.8.

### Matched controls (control)

For each of the above mentioned lung cancer patients a matched control (regarding age, gender and smoking habits) was included. These controls were taken from 600 patients who had undergone routine lung function tests in the Department of Internal Medicine and had no history or symptoms of respiratory disease. Smoking habits are matched to the lung cancer patients by classification in smoking more than 10 pack years as smokers and smoking less or never smokers as low smokers. Of these 117 patients, 100 (85.4%) had coronary heart disease, four (3.4%) had sleep apnea, three (2.5%) patients got surgery for cancer and 10 (8.5%) patients suffered from other diseases. For the unmatched analysis the NSCLC- and SCLC-matched control patients were grouped together to create a non-matched control group. The group characteristics of different subgroups are shown in Table 1AB.

### PCR

Whole EDTA-blood was used to prepare DNA using a kit provided by Qiagen (Hilden, Germany). Mapping of the intron 4 variation was performed using specific PCR. The nucleotide positions are chosen according to Pilot-Matias *et al* (1989): 161 (TGTGTGTGAGAGTGAGGGTGTAAG; antisense, position 3174–3197) and 172 (CTGGTCATCGACTACTTCCA; sense position 2561–2581). PCR was performed with 10 ng DNA as template, 0.2 units Taq polymerase (Boehringer Mannheim, Mannheim, Germany), 1.5 μl buffer 1 (17.5 mM MgCl_2_) and buffer 2 (22.5 mM MgCl_2_) 10×concentrated, (Boehringer Mannheim, Mannheim, Germany), 1 μl of specific primer (100 ng μl^−1^) 161 and 172, 1 μl dNTPs (1.25 mM each dNTP).

The PCR were performed as hot-start PCR with following cycling conditions: 30 cycles of 94°C for 30 s, 59°C for 1 min, 72°C for 1 min, and a final cycle of 5 min for 72°C. As a size marker we used cloned and sequenced intron 4 fragments as described previously (Floros *et al*, 1995; Veletza *et al*, 1996). The PCR-products and the clones were analysed by 1.5% agarose gel electrophoresis and stained with ethidium bromide.

### Statististical analysis

The statistical analysis was performed by means of the SPSS software package (Version 9.0, SPSS Inc., Chicago, IL, USA). Frequency of the SP-B variants in the respective groups (control–group, population–control, NSCLC and SCLC) was compared using the Chi-square and Fisher's Exact Test methods. Differences in the frequency of variations *vs* wild type were considered statistically significant when α was ⩽0.05 in Fisher's Exact Test. The 95% confidential interval (95% CI) depicts the confidential interval of the given odds ratios.

## RESULTS

### Description of intron 4 variants in the groups under study

**Figure 1:**
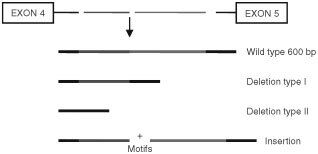
Intron 4 group variants. The invariant segment (with 11 motifs) is approximately 600 bp. The two common deletion variants are described here as type I and type II, and lack 5 or 8 motifs, respectively. The larger size variants are characterised by insertion of several motifs.

We analysed a polymorphism within intron 4 of the SP-B gene in patients with lung cancer, their matched controls and healthy individuals. This large size gene variant is characterised by deletions or insertions (Floros *et al*, 1995; Veletza *et al*, 1996), as shown schematically in Figure 1

**Figure 2:**
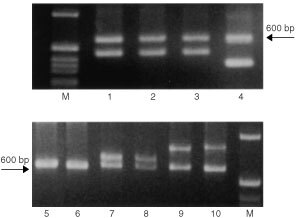
Analysis of intron 4 PCR products by agarose gel electro phoresis. M=molecular weight markers, lanes 1–4 show the type II and type I deletions respectively (lower bands). Lines 5 and 6 depict the invariant band, lanes 7–10 depict variants with different insertions (7=+2 motifs, 8=+3 motifs, 9=+6 motifs, 10=+7 motifs).

. The wild type of intron 4 consists of 11 motifs (Floros *et al*, 1995), while the variants result from gain or loss of motifs, consisting of a 20 bp conserved element, followed by a variable number of CA-repeats. The break-point of deletions or insertions does not occur in the conserved 20 bp sequences, but within the CA-repeats. The analysis showed several variants with deleted or inserted motifs that range from −8 to +7 motifs, similar to those described before (Floros *et al*, 1995). Figure 2

depicts the invariant band with a size of about 600 bp (lanes 5 and 6). The variant band is either smaller or larger. We used cloned and sequenced DNA as size markers to define the common deletions as type I or II, lacking 5 or 8 motifs, respectively. Lanes 1–4 in Figure 2 depict examples of type I or type II deletion variants, respectively, and lanes 7–10 examples of variants with different insertions.

### The frequency of intron 4 variants differs among various groups

The frequency of the intron 4 variants was analysed in 357 individuals divided in two control and two cancer patient groups. Similar to the previous studies that demonstrated an association between the SP-B intron 4 variants and acute respiratory failure in chronic obstructive pulmonary disease (COPD) (Seifart *et al*, 2002) and respiratory distress syndrome (Floros *et al*, 1995) we grouped both variants (deletions and insertions) together. The presence of the variant allele in the population and control group does not differ (9.4%, 13.0%, respectively). This frequency was increased in the SCLC and NSCLC group (17.5%, 16.9%, respectively) without reaching statistical significance (Table 2

**Table 2:**
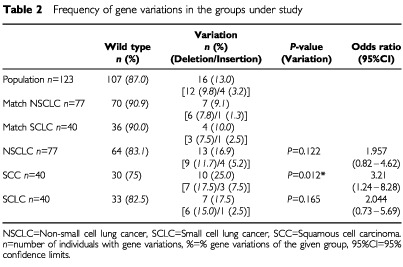
Frequency of gene variations in the groups under study

). However, with respect to the tumour histology of the NSCLC-patients, there was a high significant increase of the intron 4 variants (25.0%) in patients with squamous cell lung cancer (*P*=0.016 (Fisher's Exact Test), *P*=0.012 (Chi Square), OR=3.2, 95%CI=1.24–8.28 for the matched controls) (Table 2). Because the other histology subtypes showed very low frequencies (adenocarcinoma: 11.5%, large cell carcinoma 0.0%, other subtypes 0.0%, all together without SCC: 7.5%) it became evident that the high frequency in the SCC patients is responsible for the increase in the whole NSCLC group. Interestingly, of the three patients with SP-B gene variants belonging to the subgroup of adenocarcinoma, two were low smokers or never smokers and one smoked less than 20 p/y. In the subgroup of SCC mean p/y does not differ between those with SP-B gene variants (43.5) and those with SP-B wild type (43.9).

All samples which showed variant alleles were heterozygous, carrying one variant and one invariant allele, except for two samples, one from the SCLC group, one from the SCLC matched control, that were homozygous for the smaller size variant (deletion). The individual from the SCLC group carried both type I and type II alleles, the others were homozygous for type II deletion. Among the 47 individuals with variant alleles 23.4% (11) were due to insertions and 76.5% (36) were due to deletions. There was no significant difference in the frequency of alleles with insertions or with deletions among the different groups. From the 34 alleles with heterozygous deletions, 18 corresponded to type I, and 16 to type II deletions. Together these data indicate an association between intron 4 variability and/or its flanking loci with mechanisms that enhance susceptibility to squamous cell carcinoma of the lung.

## DISCUSSION

In the present case–control study we analysed the frequency of SP-B intron 4 variants in patients with lung cancer and demonstrated an increase of gene variants in lung cancer patients with a high significant difference in the frequency between healthy persons and patients with squamous cell lung cancer. The data suggest an association between mechanisms that enhance susceptibility to squamous cell lung cancer and the SP-B gene variants.

A number of respiratory diseases related to pathophysiological conditions of the airways have been associated with an increased risk of lung cancer. Independent of smoking behaviour, family history and occupational exposure, lung cancer risk is increased in patients with limited airway flow (Skillrud *et al*, 1986). Pulmonary surfactant is essential for normal lung function and provides several functions related to local airway conditions, such as enhancement of mucocilliary clearance, improvement of airway stability, encoating, deposition and retention of inhaled substances and particles as well as interaction with infectious agents. We focused our attention on surfactant protein B, because SP-B is necessary for the formation of the active surface film, it is essential for life (Clark *et al*, 1995) and an association of SP-B variants with acute respiratory failure in COPD was demonstrated (Seifart *et al*, 2002). Moreover, there is an association of reduced levels of SP-B observed in heterozygous SP-B knockout mice (−/+) with lung physiological abnormalities, decreased lung compliance and increased residual lung volume (Clark *et al*, 1997), as well as high susceptibility to oxidative lung injury (Tokieda *et al*, 1999).

In contrast to the alveolar surfactant that is produced by type II pneumocytes, the source of airway surfactant is less clear. It is likely that airway surfactant derives by local secretion and from the alveolar region. Epithelial cells of the tracheobronchial tree of the human have the capacity to produce SP-A, SP-B and SP-C (Phelps and Floros, 1988). Interestingly, although SP-B is synthesised by alveolar type II cells and bronchiolar epithelial cells, recent studies indicate that the function of alveolar and bronchiolar SP-B may differ (Lin *et al*, 1999b). The role of bronchiolar SP-B is currently unknown, but bronchiolar SP-B can not substitute for the function of alveolar SP-B.

The variability within intron 4 of the surfactant protein B gene is caused by the gain or loss of motifs (Floros *et al*, 1995; Veletza *et al*, 1996), consisting of a 20 bp conserved sequence followed by various dinucleotide repeats (CA-repeats). Currently, it is not known whether the intron 4 variability has an impact on SP-B gene expression or regulation. Neither is it known whether the SP-B locus itself or a linked gene contributes to squamous cell lung cancer or may be related to enhanced cancer susceptibility. However, because this variability results in large deletions or insertions, likely these alterations may compromise splicing and/or SP-B mRNA processing that in turn result in altered SP-B mRNA and/or lower amounts or impaired SP-B protein function protein. Disruption of the surfactant film and function has been demonstrated in SP-B knock-out mice (Clark *et al*, 1995, 1997), thus changes in the amount and/or the activity of SP-B may result in impaired function of surfactant. As it is known, cigarette smoking is strongly associated with the development of COPD and lung cancer. Smoking can induce alterations of the surfactant system (Finley and Ladman, 1972; Low *et al*, 1978) and these alterations may be more serious if the composition of the surfactant system is disturbed. The carcinogenic process of lung cancer is driven by the interaction of exogenous carcinogenic exposures and the cellular response, including genetic DNA repair capacity. The internal dose of carcinogens, such as tobacco smoke, the clearance and local conditions of the airways are important factors in the carcinogenesis of lung cancer, as it could be seen in p53 mutations (Suzuki *et al*, 1992; Greenblatt *et al*, 1994). Two physiological functions of the bronchiolar surfactant might play an important role in the interaction of carcinogenetic substances of the lung: The clearance of inhaled particles or substances and their encoating by surfactant.

Particles deposited on to the alveolar and airway surfaces are encoated with surfactant and displaced into the aqueous phase toward the epithelium (Allegra *et al*, 1985; Schürch *et al*, 1990). Surfactant has also been shown to enhance particle clearance by accelerating cilliary beat frequency (Kakuta *et al*, 1991; Gerber *et al*, 1995) and conditioning the viscosity of the mucus (Gerber *et al*, 1995). Surfactant coating enhances phagocytosis of inhaled particles (Stringer and Kobzik, 1996), and reduces the cytotoxity of particles and free radical activity (Cifuentes *et al*, 1995). Furthermore, it has been shown to decrease genotoxicity of non-porous particles, such as silica and asbestos (Liu *et al*, 1994) and exogenous surfactant treatment improves sputum mucociliary transportability and lung function in patients with chronic bronchitis (De Sanctis *et al*, 1994; Anzueto *et al*, 1997). Disturbance of these functional properties may mediate local airway conditions and result in prolonged exposure and impaired interaction with carcinogens. Therefore, the individual variability in the response to environmental mutagens may be related to local factors mediated by surfactant function. This hypothesis might be supported by the observation that the association between exogenous exposure to carcinogens has been shown strongest for squamous cell carcinoma of the lung (Breslow *et al*, 1954; Weiss *et al*, 1972) and the relative risk with increasing duration of exposure rise fastest in this type of lung cancer. But, as SCLC is also related to tobacco smoke and other exogenous substances, the differences in SP-B variants in SCC and SCLC might be due to statistical reasons because of small groups. Otherwise it might be due to the different cell type of origin and differences in developing pathways, as SCC arises from progression of metaplasia and dysplasia, while SCLC does not in the same way. Although characteristic patterns of DNA changes are caused by exogenous mutagens in both lung cancer types for several molecular genetic changes, such as G-protein pathways and MYC genes, differences between SCC and SCLC have been shown (Greenblatt and Harris, 1995). Currently, it is not known why there is a different mutational spectrum in those cancer types. For adenocarcinoma, as this lung cancer type is not strongly related to exogenous mutagens, the SP-B frequency is similar to the controls. For the other types of lung cancer derives from small numbers of individuals, the SP-B frequency could not be evaluated.

In summary, following investigation of differences in the frequency of the SP-B intron 4 variants among various control and lung cancer groups, we observed an association between this polymorphism and a subgroup of NSCLC, the squamous cell lung cancer. Although further studies are needed to determine the impact on protein expression, and to determine whether the SP-B locus itself contributes to the disease, it suggests that these gene variations are associated with mechanisms of enhanced lung cancer susceptibility. The effects of smoking are undoubtedly predominant, but changes in airway conditions related to SP-B gene variants or flanking loci may work as confounding risk factors to squamous cell carcinoma.
